# Cabotegravir pharmacokinetics in Asians with and without HIV 

**DOI:** 10.5414/CP204849

**Published:** 2025-07-02

**Authors:** Kelong Han, Ahmed A. Abulfathi, Rashmi S. Mehta, Romina A. Nand, Mark A. Marzinke, Raphael J. Landovitz, Ronald D. D’Amico, Alex R. Rinehart, William R. Spreen, Susan L. Ford

**Affiliations:** 1 GlaxoSmithKline, Collegeville,; 2 Certara USA, Inc, Radnor, PA,; 3 GlaxoSmithKline, Durham, NC, USA,; 4 GlaxoSmithKline, Sydney, Australia,; 5 Johns Hopkins University School of Medicine, Baltimore, MD,; 6 Center for Clinical AIDS Research and Education, David Geffen School of Medicine, University of California, Los Angeles, CA, and; 7 ViiV Healthcare, Durham, NC, USA; * Contributed equally

**Keywords:** cabotegravir, long-acting, Asian, HIV, pharmacokinetics

## Abstract

Objective: Cabotegravir is approved for HIV treatment (with rilpivirine) and prevention. The established cabotegravir population pharmacokinetic (PPK) model included 1.2% Asian participants. We aimed to compare cabotegravir pharmacokinetics between Asian and non-Asian populations and across Asian countries. Materials and methods: Cabotegravir concentrations were collected from Asian participants in phase 1 and 3 studies. The applicability of the PPK model to Asian populations was validated by predicting the observed concentrations not included in model-building. Cabotegravir post-hoc pharmacokinetic parameters (long-acting absorption rate constant, weight-normalized apparent clearances and volumes of distribution) and exposures (trough and peak concentrations) following monthly and every-2-month regimens were estimated by fitting the PPK model to observed data. Non-Asian participants from the previous PPK dataset (1,697 males; 564 females) were used as comparator. Cabotegravir exposures in Asian and non-Asian were compared via simulations. Results: 2,034 cabotegravir concentrations were collected from 162 Asian males (assigned male at birth) in China (n = 47), Japan (n = 17), Korea (n = 25), Thailand (n = 53), and Vietnam (n = 20), and 35 concentrations from 2 Asian females (assigned female at birth) in Korea. Cabotegravir pharmacokinetic parameters were similar between Asian and non-Asian participants. Cabotegravir exposures in Asian populations largely overlapped with but tended to be higher than non-Asian populations, suggesting similar efficacy. Cabotegravir exposures in Asian and non-Asian populations remained below the safety threshold, suggesting similar safety profiles. Cabotegravir pharmacokinetic parameters and exposures were similar across Asian countries. Conclusion: No dose adjustment is recommended for Asian populations with and without HIV. Cabotegravir pharmacokinetic data from any Asian country/region may guide pharmacokinetic evaluation and regulatory considerations across Asian regions.

**What is known about this subject**

- Cabotegravir (CAB) long-acting (LA) plus rilpivirine LA administered monthly (QM) or once every 2 months (Q2M) via intramuscular (IM) gluteal injections is a complete LA regimen approved for maintenance of HIV-1 virologic suppression in people with HIV. CAB LA as a single-agent administered Q2M is approved for HIV-1 pre-exposure prophylaxis (PrEP).
- Cabotegravir clinical data for Asian populations is limited in the public domain, which potentially leaves clinical practice in Asian countries underinformed, especially considering that CAB pharmacokinetics (PK) is associated with factors that may differ between Asian and non-Asian populations.

**What the study adds**

- Cabotegravir exposures in the Asian population largely overlapped with but tended to be higher than non-Asian population, suggesting that it is reasonable to anticipate comparable efficacy between Asian and non-Asian populations.
- Cabotegravir exposures in the Asian population remained well below the safety threshold, suggesting that it is reasonable to anticipate comparable safety profiles between Asian and non-Asian populations. Therefore, no dose adjustment is recommended for the Asian population with and without HIV.
- Cabotegravir PK parameters and exposures were comparable across Asian countries, supporting the notion that PK data from any Asian country/region may be used to guide CAB PK evaluation and regulatory considerations, thereby potentially avoiding unnecessary PK studies for future clinical development of CAB.

## Introduction 

Cabotegravir (CAB) is an HIV integrase strand transfer inhibitor [1]. CAB long-acting (LA) plus rilpivirine (RPV) LA, administered monthly or every 2 months via intramuscular gluteal injections (Cabenuva, ViiV Healthcare, Research Triangle Park, NC, USA and Vocabria, ViiV Healthcare, Research Triangle Park, NC, USA), is the first and currently the only complete LA regimen approved for maintaining HIV-1 virologic suppression. CAB LA alone, administered every 2 months (Apretude, ViiV Healthcare, Research Triangle Park, NC, USA), is the first and currently the only LA injectable approved for HIV-1 pre-exposure prophylaxis (PrEP). 

CAB clinical development remains active [2]. In 2018, an estimated 5.1 – 7.1 million individuals lived with HIV in Asia and the Pacific, and only 49% were virologically suppressed (https://www.unaids.org/sites/default/files/media_asset/2019-global-AIDS-update_asia-pacific_en.pdf). Publicly available CAB clinical data for Asian populations is limited, potentially leaving clinical practice in Asian countries underinformed, especially considering that CAB pharmacokinetics (PK) is associated with intrinsic and extrinsic factors that may differ between Asian and non-Asian populations. Firstly, CAB apparent clearances and volumes of distribution increase with body weight, and body weight in Asian countries is generally lower than in non-Asian countries such as the United States and Spain (https://www.worlddata.info/average-bodyheight.php), the top 2 countries ranked by CAB clinical trial enrollment. Secondly, CAB clearance is 17.4% higher in current smokers than non-smokers [1]. Male smoking rates are higher in China (48%), Japan (33%), Korea (38%), Thailand (43%), and Vietnam (43%) than in non-Asian countries such as the United States (31%) and Spain (29%), while female smoking rates are lower in China (2%), Japan (11%), Korea (6%), Thailand (3%), and Vietnam (2%) than in non-Asian countries such as United States (19%) and Spain (27%) (https://www.worldpopulationreview.com/country-rankings/smoking-rates-by-country). Finally, UGT1A1 is the primary hepatic elimination pathway of CAB, and UGT1A1 polymorphisms (e.g., *6 variant common in East Asians) are associated with a clinically non-significant increase in CAB exposure [3]. 

The established CAB population PK (PPK) model [1], based on 23,926 plasma concentrations from 1,647 adults with (72%) and without (28%) HIV, included only 20 Asian participants (8 from Japan and 12 from Korea, all assigned male at birth), representing 1.2% of the dataset. Race and HIV status were not significant covariates. Long-acting absorption rate constant (KA_LA_) was 50.9% lower in females than males (sex assigned at birth). The model was validated by adequately predicting 5,097 concentrations from 647 participants who were not included in the model-building dataset [1], only 15 of whom (2.3%) were Asian (Korea). 

The objectives of this work were to assess the adequacy and predictive performance of the CAB PPK model in Asian populations, and to compare CAB PK parameters and exposures between Asian and non-Asian populations and across Asian countries. 

## Materials and methods 

Asian participants/population were defined as participants of Asian ancestry living in Asian countries. CAB concentrations were collected from phase 1 and 3 studies. Missing smoking status was imputed as non-smoker. As sensitivity analysis, all analyses were repeated by imputing current smoker. The applicability of the PPK model to Asian populations was validated using prediction-corrected visual predictive checks with 1,000 replications to predict the observed concentrations in Asian populations not included in model-building. 

CAB post-hoc PK parameters and exposures following monthly and every-2-month regimens were estimated by fitting the PPK model to observed data. Exposures included trough concentration (C_tau_) following the first injection (C_tau_-1), C_tau_ at steady state (C_tau_-SS) and peak concentration (C_max_) at steady state (C_max_-SS). Non-Asian participants from the previous PPK dataset [1] (1,697 males, 564 females) served as comparators. Post-hoc PK parameters and exposures were compared between Asian and non-Asian participants and across Asian countries. Median C_max_-SS was compared to a safety threshold of 13.1 µg/mL, defined as the median C_max_ observed following daily oral CAB 60 mg (twice the approved CAB oral dose of 30 mg) in study LAI116482 (NCT01641809), which was the highest C_max_ ever observed in CAB long-term studies in adults and not associated with any toxicity. 

## Results 

A total of 2,034 CAB plasma concentrations were collected from 162 Asian males (China n = 47; Japan n = 17; Korea n = 25; Thailand n = 53; Vietnam n = 20) and 35 concentrations from 2 Asian females (Korea). Body weight and body mass index (BMI) were similar across the 5 Asian countries, but lower in Asian than in non-Asian males. Median (range) body weight was 66.4 kg (42.5 – 103) in Asian males and 78.1 kg (49.4 – 168) in non-Asian males. Median (range) BMI was 23.0 kg/m^2^ (15.1 – 40.5) in Asian males and 25.1 kg/m^2^ (16.6 – 69.5) in non-Asian males. The 2 Asian females had body weights (66.8 and 67 kg) and BMIs (25.5 and 27.9 kg/m^2^) similar to the median body weight (70 kg) and BMI (26.4 kg/m^2^) in non-Asian females. 

The CAB PPK model adequately described and predicted the observed CAB plasma concentration-time profiles in each Asian country, with 90% of observed concentrations within the 90% prediction interval. 

**Figure 1:**
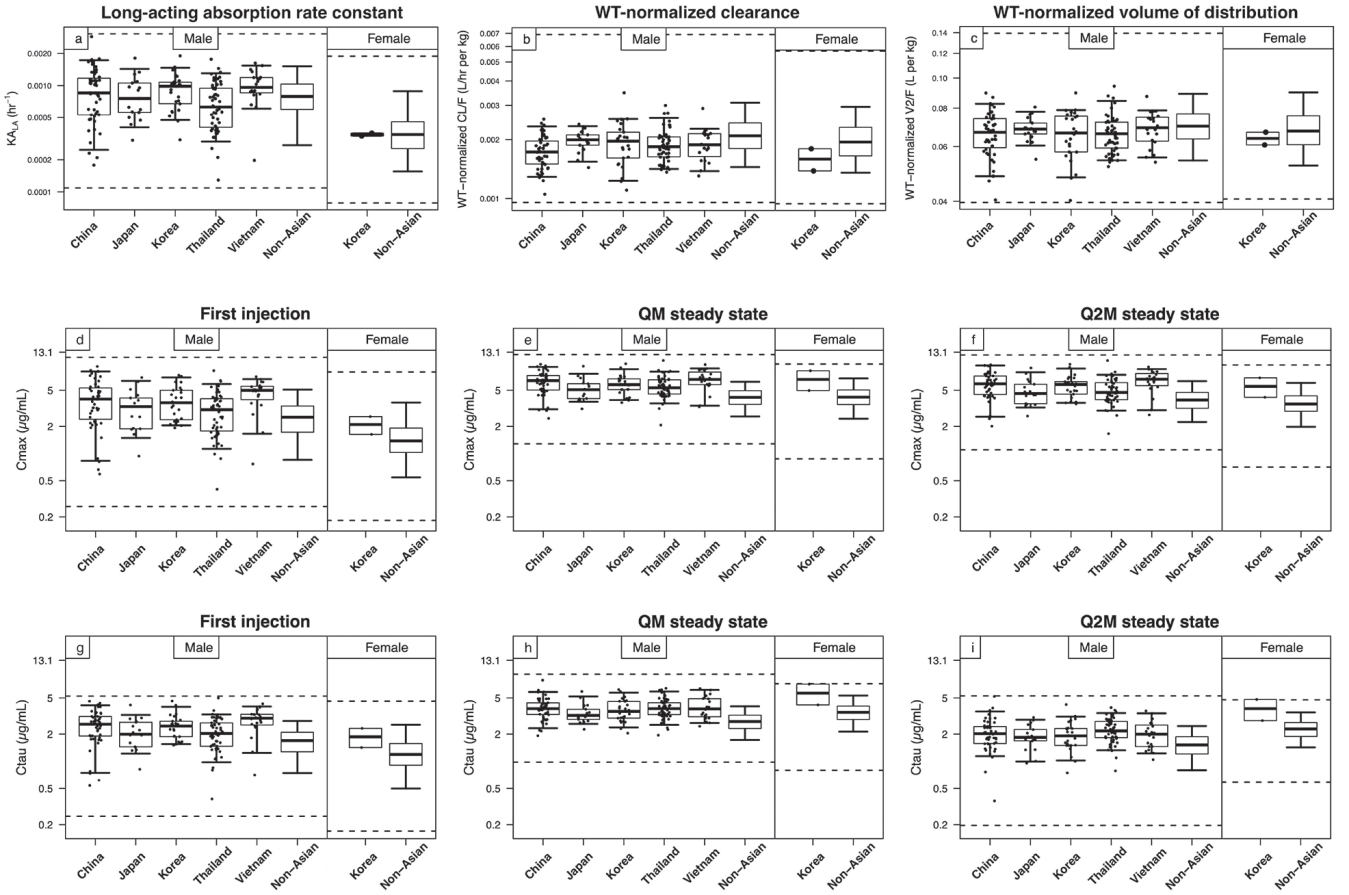
Comparisons of cabotegravir post-hoc estimates of pharmacokinetic parameters (a – c) and plasma exposures (d – i) in Asian and non-Asian participants. Pharmacokinetic parameters included (a) KA_LA_, (b) weight-normalized CL/F, and (c) weight-normalized V2/F. Exposures included C_max_ (a, b, c) and C_tau_ (d, e, f) following the initiation injection (a, d), at steady state of QM injections (b, e), and at steady state of Q2M injections (c, f). Boxes represent interquartile ranges; solid horizontal lines represent medians; error bars represent 5^th^ and 95^th^ percentiles; solid circles represent individual values (individual values for non-Asian participants are not displayed for cosmetic reason). Horizontal dashed lines represent minimum and maximum values in non-Asian participants. CL/F = apparent central clearance; C_max_ = maximum concentration; C_tau_ = trough concentration; KA_LA_ = long-acting absorption rate constant; Q2M = once every 2 months; QM = once monthly; V2/F = apparent central volume of distribution; WT = body weight.

Post-hoc estimates of KA_LA_, weight-normalized clearance, and central volume of distribution were similar between Asian and non-Asian participants (Figure 1a – c), and all Asian values fell within the non-Asian range. 

CAB exposures largely overlapped between Asian and non-Asian populations with all Asian values falling within the non-Asian range despite a trend towards higher exposures in Asian populations (Figure 1d – i), suggesting similar efficacy for Asian and non-Asian populations. CAB exposures in both populations remained below the safety threshold of 13.1 µg/mL (Figure 1d – i), suggesting similar safety profiles for Asian and non-Asian populations. 

CAB PK parameters (Figure 1a – c) and exposures (Figure 1d – i) were similar across Asian countries. 

Similar results were obtained by imputing missing smoking status as current smoker. 

## Discussion 

The trend towards higher CAB exposures in Asian than in non-Asian populations is likely due to lower body weight and higher prevalence of UGT1A1*6 variant in Asians, both reducing clearance. However, this trend is unlikely to be clinically significant. Firstly, CAB exposures largely overlapped between Asian and non-Asian populations, with all Asian values within the non-Asian range. Furthermore, higher C_tau_ is not expected to compromise efficacy and has been associated with higher efficacy of CAB + RPV LA for treatment [4]. Therefore, CAB efficacy in Asian populations is expected to be similar to that observed in non-Asian populations, for which efficacy has been extensively demonstrated in phase 3 studies. Finally, CAB exposures in Asian populations remained well below the pre-defined safety threshold of 13.1 µg/mL. Therefore, no dose adjustment is recommended for the Asian population with or without HIV. 

This analysis has limitations. Firstly, PK data from Asian females were limited, with only 2 Asian females from Korea. To address this, simulations were performed to compare CAB exposures between Asian and non-Asian females, and results aligned with observations from Asian males and the 2 Asian females, though more real-world data in Asian females would strengthen conclusions. Additionally, UGT1A1 genotype was not measured, but this is unlikely to impact the conclusions, because its effect on CAB concentrations is considered clinically insignificant [3]. Finally, missing smoking status was imputed as non-smoker, but this is unlikely to impact the conclusions, because sensitivity analyses using alternative imputations yielded similar results. 

## Conclusion 

CAB exposures in the Asian population largely overlapped with but tended to be higher than in the non-Asian population, suggesting comparable efficacy between Asian and non-Asian populations. CAB exposures in the Asian population remained well below the safety threshold, suggesting comparable safety profiles between Asian and non-Asian populations. No dose adjustment is recommended for the Asian population with or without HIV. CAB PK parameters and exposures were comparable across Asian countries, supporting the notion that PK data from any Asian country/region may be used to guide CAB PK evaluation and regulatory considerations across Asian regions, thereby potentially avoiding unnecessary PK studies for future clinical development of CAB. 

## Ethnicity statement 

This work and conclusions focus on the Asian population in comparison with the non-Asian population and across Asian countries. 

## Acknowledgments 

This study was funded by ViiV Healthcare. The authors thank the study participants and their families and caregivers, the investigators and site staff who participated in the studies 206898, FLAIR, ATLAS, ATLAS-2M, and HPTN 083, and the ViiV Healthcare and GSK study team members. 

## Authors’ contributions 

All listed authors meet the criteria for authorship set forth by the journal and by the International Committee for Medical Journal Editors. All listed authors have significantly contributed to, reviewed, and approved the final submitted version of the manuscript. 

KH, AAA, RSM, RAN, MAM, RJL, RDD, ARR, WRS and SLF contributed to the conception, design, data acquisition, data analysis, and data interpretation for the work. KH, AAA, RSM, RAN, MAM, RJL, RDD, ARR, WRS and SLF contributed to drafting, reviewing and approving the manuscript. 

## Funding 

This is a meta-analysis supported by ViiV Healthcare. There was no external funding. 

## Conflict of interest 

KH, RSM, RAN, and SLF receive salary from GSK and hold stocks of GSK. RDD, ARR, and WRS receive salary from ViiV Healthcare and hold stocks of GSK. AAA receives salary from Certara and hold stocks of Certara. MAM receives salary from Johns Hopkins University. RJL receives salary from University of California, Los Angeles.
